# Reporting quality of statistical methods in surgical observational studies: protocol for systematic review

**DOI:** 10.1186/2046-4053-3-70

**Published:** 2014-06-28

**Authors:** Robert Wu, Peter Glen, Tim Ramsay, Guillaume Martel

**Affiliations:** 1 Department of Surgery and Ottawa Hospital Research Institute, University of Ottawa, 501 Smyth Rd, CCW 1667, K1H 8L6 Ottawa, ON, Canada

**Keywords:** Medicine, Observational studies, Statistics, Surgery, Systematic review

## Abstract

**Background:**

Observational studies dominate the surgical literature. Statistical adjustment is an important strategy to account for confounders in observational studies. Research has shown that published articles are often poor in statistical quality, which may jeopardize their conclusions. The Statistical Analyses and Methods in the Published Literature (SAMPL) guidelines have been published to help establish standards for statistical reporting.

This study will seek to determine whether the quality of statistical adjustment and the reporting of these methods are adequate in surgical observational studies. We hypothesize that incomplete reporting will be found in all surgical observational studies, and that the quality and reporting of these methods will be of lower quality in surgical journals when compared with medical journals. Finally, this work will seek to identify predictors of high-quality reporting.

**Methods/Design:**

This work will examine the top five general surgical and medical journals, based on a 5-year impact factor (2007–2012). All observational studies investigating an intervention related to an essential component area of general surgery (defined by the American Board of Surgery), with an exposure, outcome, and comparator, will be included in this systematic review. Essential elements related to statistical reporting and quality were extracted from the SAMPL guidelines and include domains such as intent of analysis, primary analysis, multiple comparisons, numbers and descriptive statistics, association and correlation analyses, linear regression, logistic regression, Cox proportional hazard analysis, analysis of variance, survival analysis, propensity analysis, and independent and correlated analyses. Each article will be scored as a proportion based on fulfilling criteria in relevant analyses used in the study. A logistic regression model will be built to identify variables associated with high-quality reporting. A comparison will be made between the scores of surgical observational studies published in medical versus surgical journals. Secondary outcomes will pertain to individual domains of analysis. Sensitivity analyses will be conducted.

**Discussion:**

This study will explore the reporting and quality of statistical analyses in surgical observational studies published in the most referenced surgical and medical journals in 2013 and examine whether variables (including the type of journal) can predict high-quality reporting.

## Background

Evidence-based medicine provides an important framework for clinical decision making [1]. The utilization of evidence-based medicine in surgery requires a clinician to find the best available evidence and to critically appraise the validity and usefulness of the information [2]. Unfortunately, clinical evidence in the literature is of unequal quality. While well-conducted clinical trials may provide the highest level of evidence, many clinical questions are difficult to answer with trials. This is often due to side effects of interventions and various ethical dilemmas [3]. Surgical trials, in particular, face the additional challenge of clinical heterogeneity associated with varied techniques, perioperative care, and surgeon and supporting staff learning curves during the course of a study [4-6]. As a result, surgical trials have been few and far between, with surgical decision making remaining heavily influenced by a large body of observational literature.

In order to address potential confounders associated with their design, observational studies typically use statistical methods to compare study groups as well as to establish the association between intervention and outcome. Despite a variety of possible statistical manipulations, empirical work has shown that the effects of interventions in observational studies can be different in direction and magnitude when compared to that of randomized controlled trials [7,8]. This discrepancy can be potentially attributed to the variable quality of statistical methodology used in observational studies. As a consequence, the statistical methodology can clearly influence our ability to evaluate whether confounding has been sufficiently accounted for in a given study. It is therefore important to be comprehensive and transparent with statistical reporting when publishing observational studies.

Empirical research evidence would suggest that a significant proportion of articles are flawed in the application and reporting of statistical methods [9-11]; errors could be severe enough to jeopardize the conclusion reached by the authors [12]. Many of the articles with noticeable statistical deficiencies are found in highly-referenced clinical journals [13,14]. For instance, one study examined 100 papers in cancer journals and found that missing data may be found in 96% of the articles, with only 10% having explored the impact of such missing data on outcomes [13]. Indeed, it is known that missing data may introduce bias leading to under- and over-estimation of association between the exposure and outcome [15]. The amount of missing data also serves as a measure of study quality. Hence, it is important for the authors to provide sufficient information on missing data to enable accurate judgment of study quality. As Lang et al. have argued, such problems of poor statistical reporting concerning basic statistics are long-standing and widespread, but often go undetected [16].

In 2008, the Strengthening the Reporting of Observational Studies in Epidemiology (STROBE) statement was published to standardize the overall quality of reporting of observational studies [17]. The STROBE statement, however, focuses more on general quality assessment and is limited to addressing the specific statistical adjustments employed by authors. To complement the STROBE guidelines with more specific criteria, the EQUATOR (Enhancing the QUAlity and Transparency of health Research) network published the Statistical Analyses and Methods in the Published Literature (SAMPL) guidelines to assess the quality of statistical reporting based on the type of analysis performed by authors [18].

Given that surgical decision making continues to rely heavily upon observational studies and that the validity of such work depends in large part upon adequate statistical analysis, it becomes particularly important to examine the quality and reporting of such analyses. As such, the objective of the proposed systematic review is to assess and compare the quality and reporting of statistical methods in surgical observational studies published in the highest-impact general surgical and general medical journals in 2013. More specifically, this work will adapt and utilize a tool to evaluate the quality and reporting of statistical analysis in observational studies, evaluate the risk of statistical deficiencies, compare the quality and reporting of statistical analysis in studies published on surgical topics in surgical and medical journals, and identify factors associated with high-quality reporting. This work’s primary hypothesis is that reporting of statistical methods will be generally poor for all surgical observational studies, and that reporting within the highest referenced medical journals will be superior to that published in surgical journals. The basis for this hypothesis resides with the knowledge that general medical journals tend to have much higher impact factors than surgical journals [19], while evidence suggests that higher impact factors may be associated with higher methodological quality [20].

It can be expected that this work will be significant in defining the degree of deficiencies in the quality and reporting of statistical methods in surgical observational studies, and may be used to drive improvements.

## Methods

The framework for this study will be that of a systematic review of all observational studies pertaining to general surgical topics published in leading medical and surgical journals, where such studies are compared and analyzed for statistical quality and reporting.

1. Study inclusion

a) Types of journals:

▪ General medical and general surgical journals, without a specific sub-specialty focus.

▪ Top five general medical journals and top five general surgical journals based on 5-year impact factors.

b) Types of studies to be included:

▪ Studies published in 2013.

▪ All observational studies, including before-and-after studies, cohort studies, case-control studies, and cross-sectional studies with an exposure, outcome, and comparator group.

▪ Any investigation topic related to an essential component area of general surgery, as defined by the American Board of Surgery (alimentary tract, abdomen and its content, endocrine system, head and neck surgery, pediatric surgery, surgical critical care, surgical oncology, trauma/burns, vascular surgery) [21].

c) Types of studies to be excluded:

▪ Systematic reviews, meta-analyses, review articles, randomized controlled trials, quasi-randomized trial, other interventional studies, case reports.

▪ Studies on the topics of surgical education, diagnostic tests, quality of programs, or not otherwise directly related to clinical care.

d) Types of participants:

▪ All studies of humans, including both children and adults, will be included.

e) Types of publications to be included:

▪ Original articles only.

▪ Published abstracts and unpublished data will not be included.

2. Search strategy and study selection

a) Journals selection:

▪ The five general medical and general surgical journals with the highest 5-year impact factor for 2012 (according to ISI Web of Knowledge Journal Citation Reports [19].

▪ General medical journals: *New England Journal of Medicine*, *Lancet*, *Journal of the American Medical Association*, *PLoS Medicine*, and *Annals of Internal Medicine*

▪ General surgical journals: *Annals of Surgery*, *British Journal of Surgery*, *Archives of Surgery/JAMA Surgery*, *Journal of the American College of Surgeons*, and *Surgery*

b) Study selection:

▪ All papers published in 2013 in the relevant journals will be identified.

▪ All studies will be identified by hand searching the journals.

▪ Two reviewers will screen one month for each journal to validate the screening strategy (RW and PG). If there is greater than 90% agreement, the search strategy will be considered valid. If less than 90% agreement, the search will be repeated for a second month in each journal until 90% agreement is reached. All conflicts will be resolved with the senior author (GM).

▪ When the search is validated, all remaining studies within the relevant journals will be screened based on titles and abstracts for inclusion by one reviewer (RW or PG).

▪ Potentially relevant studies will be retrieved in full text and the final list of included studies will be generated based on inclusion and exclusion criteria by two reviewers (RW, PG).

▪ Disagreements in the study selection process will be resolved by consensus with the senior author (GM).

▪ Reasons for exclusion from the review will be identified and recorded.

3. Outcomes

a) Primary outcome:

▪ The primary outcome will be the quality of statistical reporting for individual items within the instruments. In addition, a composite score will be generated for each study, representing the proportion of items that have been adequately fulfilled within the relevant statistical domains used in a given study.

▪ A comparison of scores between surgical observational studies published in surgical and medical journals will be considered to be a primary outcome.

b) Secondary outcome:

▪ Frequency and type of statistical tests used in medical and surgical journals will be compared.

▪ Given the statistical tests used, the most often reported and missed criteria will be identified.

▪ Among statistically significant study results, the items that are more likely to be reported/omitted will be identified.

▪ Potential correlation between impact factor and overall/item-wise score.

4. Study quality and assessment

a) Statistical quality:

▪ The quality of statistics within individual studies will be assessed according to 11 domains, each comprising specific criteria (see Appendix 1).

▪ Quality assessment criteria were adapted from the SAMPL guidelines [18].

▪ The propensity score criteria were generated based on the work of Austin et al. [22].

▪ A draft outline of essential elements related to statistical quality was first generated; disagreements were resolved based on consensus. The criteria list was then further revised in collaboration with a senior statistician and methodologist (TR). The final instrument was chosen to represent a necessary set of criteria to evaluate statistical quality and reporting in observational studies.

b) Statistical assessment:

▪ The instrument will be applied independently to each study by two reviewers (RW, PG).

▪ For each study, the reviewer assessments will be compared for discrepancies and disagreements will be resolved based on consensus and discussion with the senior authors (GM and/or TR).

▪ Given the wide variability in the type of statistical analyses that can be carried out in observational studies, it is understood that not all 11 domains of quality/reporting will be applicable for each study.

▪ Study authors will be contacted selectively to provide missing data or additional details of their statistical analyses.

5. Data collection and analysis

a) Data extraction and management:

▪ A data extraction form has been designed based on input from all authors. This abstraction form was adapted from the SAMPL guidelines with modifications to reflect minimal and high impact reporting standards that need to be available to appraise the validity of an observational study. The form was first drafted by two authors (GM and RW) and modified by a senior statistician (TR). Given that the tool contains items derived from an existing guideline, it is believed the validity of the tool is retained.

▪ All types of statistical analyses within each primary study will be identified.

▪ Two reviewers (RW and PG) will independently extract data and any unresolved discrepancies will be resolved by the senior author (GM).

▪ Abstracted data will be collected within spreadsheets.

b) Data analysis:

▪ All collected data will be analyzed.

▪ The proportion of studies fulfilling individual items within the instruments will be computed. In addition, a composite score will be generated for each study, representing the proportion of items that have been adequately fulfilled within the relevant statistical domains used in a given study.

▪ The primary outcome will be computed for each study and its mean/median and measure of variability will be calculated.

▪ Data pertaining to medical and surgical journals will be compared and contrasted using a χ^2^ test.

▪ Variables associated with high-quality reporting of statistical analysis will be identified using a logistic regression model. The cohort of studies will first be dichotomized on the basis of the 75th percentile of the proportion of fulfilled criteria. This arbitrary cutoff is chosen, as it reflects the 25% of papers that will present the highest proportion of fulfilled criteria. All variables with a *P* <0.2 on univariate comparison between high- and low-quality reporting will be included in the model. The following minimal set of variables will be compared: journal name, impact factor, medical/surgical journal, continent of origin, sample size, disease category, type of exposure, and type of primary analysis. Interaction between variables and colinearity will be checked.

▪ Secondary outcomes will be compared both quantitatively and by generating a qualitative synthesis.

c) Subgroup analyses:

▪ Analysis of the subgroup of studies with higher reported strength of association (relative risk of >2 or <0.5) between exposure and outcome (GRADE assessment tool) [23].

d) Sensitivity analysis:

a) The two medical journals with the fewest published surgical observational studies, and the two surgical journals with the fewest published surgical observational studies will be removed and the analysis repeated. We hypothesize that eliminating those journals with a low publication rate will improve the overall quality of reporting.

## Discussion

This study will examine the quality and reporting of statistical methodology in surgical observational studies. It is expected that significant problems with statistical methodology will be identified, and that this problem will be more pronounced within studies published in general surgical journals. This work is important, as it will shed a critical light onto the most common type of surgical research performed to date.

The main limitation of the study is the abstraction tool derived from the SAMPL guideline, which was not constructed for scoring statistical quality. The individual items within the guideline are nonetheless important elements to understand the validity of a published study. While the instrument that is proposed in this work is not validated, it is important to emphasize that no validated instrument currently exists (including SAMPL), and as such it can be argued that this is an appropriate first step in examining this topic. Furthermore, this study focuses upon the most referenced journals to reflect the status of current statistical reporting and not all journals are presented. However, the highest impact journals have the utmost visibility in the surgical literature and are likely more relied upon by surgeons to inform practice.

The findings of this review may provide an opportunity for surgical researchers and journal editors to improve the quality of statistical analyses being performed, as well as to call for improved and more transparent reporting of statistical methodology.

## Appendix 1. Criteria for assessment of statistical quality

1. Intent of analysis

a) Is there evidence of *a priori* definition of primary endpoint, reflected in any of the following?

• Protocol use

• Explicit statement: there is an *a priori* objective

• Sample size calculation

• If subgroup analyses were used, acknowledge the use of:

◦ Subgroup analysis/sensitivity

◦ Multiple comparisons

◦ Statistical methods/tests for subgroup comparisons

2. Preliminary analysis

a) Identify any statistical procedures used to modify raw data before analysis (e.g., transformation of data to move closer to normal distribution, creating ratios or other values, collapsing continuous into categorical data, or combining categories)

3. Methodological principles and primary analysis

a) Identification of a smallest clinically important difference for the primary outcome

b) For primary endpoint, report distribution type:

i) Normal distribution: report as mean and SD

ii) Non-normal: report as median and interpercentile range, range or both

4. Numbers and descriptive statistics

a) Report total sample and per group

b) Report missing/loss to follow-up and how the missingness is statistically accounted for (e.g., imputation, sensitivity analysis)

5. Association analyses

a) Report values of coefficients and confidence intervals if a measure of association is used

6. Correlation analyses

a) Report value of correlation coefficient and confidence interval for the coefficient

7. How was confounding/bias accounted for?

1. Matching (matching analysis, propensity matching)

2. Stratification

3. Standardization

4. Multivariate analysis

a) Linear

b) Logistic

c) Cox

d) ANOVA

e) Propensity/Instrumental variable

8. Linear regression analysis/logistic regression/Cox proportional hazard

a) Identify all variables used in the comparison (what is the ratio of covariates to events?)

b) Confirm that the assumptions of the specific type of regression analysis have been met, state how each assumption was checked

c) Report how any missing data were treated in the analysis

d) Specify how the explanatory variables that appear in the final model were chosen

e) Specify whether all potential explanatory variables were assessed for colinearity

f) Specify whether all potential explanatory variables were tested for interaction

g) Specify whether time-dependent covariates were examined/used (Cox regression)

h) Provide a measure of the model’s goodness of fit

9. ANOVA/ANCOVA

a) Identify all variables used in the comparison

b) Confirm that the assumptions of the analysis have been met, state how each assumption was checked

c) Report how any missing data were treated in the analysis

d) Specify whether all potential explanatory variables were tested for interaction

e) Report the results of the ANOVA in a table, *P* value for each explanatory variable, test statistics

f) Provide a measure of the model’s goodness-of-fit

10. Survival analysis

a) Identify dates or events marking the beginning and the end of the time period analyzed

b) Identify circumstances when data were censored

c) Identify methods used to estimate survival rates

d) Confirm that assumptions of survival analysis have been met

11. Propensity analyses

a) Describe how propensity score was specified

i) Describe how variables were selected for consideration of inclusion in the propensity score model

ii) Describe how the propensity score model was formulated

## Abbreviations

SAMPL: Statistical Analyses and Methods in the Published Literature Guidelines; STROBE: Strengthening the Reporting of Observational Studies in Epidemiology Statement.

## Competing interests

The authors declare that they have no competing interests.

## Authors’ contributions

All authors read and approved the final manuscript. RW: design and conception of the study, drafting of manuscript. PG: design and conception of the study, drafting of manuscript. TR: design of the study, critical revision of manuscript. GM: conception and design of the study, clinical expertise, drafting of manuscript, critical revision of manuscript, project supervision. All authors read and approved the final manuscript.

## Acknowledgements

None.
